# AccPbFRET: An ImageJ plugin for semi-automatic, fully corrected analysis of acceptor photobleaching FRET images

**DOI:** 10.1186/1471-2105-9-346

**Published:** 2008-08-19

**Authors:** János Roszik, János Szöllősi, György Vereb

**Affiliations:** 1 Department of Biophysics and Cell Biology, Research Center for Molecular Medicine, University of Debrecen, Debrecen, Hungary

## Abstract

**Background:**

The acceptor photobleaching fluorescence resonance energy transfer (FRET) method is widely used for monitoring molecular interactions in cells. This method of FRET, while among those with the simplest mathematics, is robust, self-controlled and independent of fluorophore amounts and ratios.

**Results:**

AccPbFRET is a user-friendly, efficient ImageJ plugin which allows fully corrected, pixel-wise calculation and detailed, ROI (region of interest)-based analysis of FRET efficiencies in microscopic images. Furthermore, automatic registration and semi-automatic analysis of large image sets is provided, which are not available in any existing FRET evaluation software.

**Conclusion:**

Despite of the widespread applicability of the acceptor photobleaching FRET technique, this is the first paper where all possible sources of major errors of the measurement and analysis are considered, and AccPbFRET is the only program which provides the complete suite of corrections – for registering image pairs, for unwanted photobleaching of the donor, for cross-talk of the acceptor and/or its photoproduct to the donor channel and for partial photobleaching of the acceptor. The program efficiently speeds up the analysis of large image sets even for novice users and is freely available.

## Background

Fluorescence resonance energy transfer (FRET) is a powerful technique that can be applied to study nanoscale intra- and intermolecular events and interactions of molecules in situ in biological systems [1]. In assessing FRET, fluorescence of a spectrally matched donor and acceptor dye pair can be measured to reveal the radiationless transfer of excitation energy from the donor to the acceptor, in the case that their dipoles are properly oriented and the two are in spatial proximity (usually at a distance of 1–10 nm) [2]. This latter phenomenon is the basis of the popularity of FRET in biology: The distance over which FRET occurs is small enough to characterize the proximity of possibly interacting molecules, under special circumstances it even provides quantitative data on exact distances, and, additionally, information on the spatial orientation of molecules or their domains. Hence the very apropos term from Stryer, who equaled FRET to a "spectroscopic ruler" [3]. FRET can be measured both in microscopic imaging and in flow cytometry. While flow cytometric FRET (FCET) carries the advantage of examining large cell populations in a short time, microscopic approaches have the ability to provide subcellular detail and the possibility to correlate FRET values with other biological information gained from fluorescent labeling, on a pixel by pixel basis [4]. In a review about FRET imaging, Jares-Erijman and Jovin classified 22 different approaches to quantifying FRET in a systematic way. The techniques fall in two major groups: most of them are based on donor quenching and/or acceptor sensitization, and a few on measuring emission anisotropy of either the donor or the acceptor [5]. In the practice of cell biology, ordinary confocal microscopy is now broadly available, and brings three quantitative FRET approaches within close reach. These are the various ratiometric approaches, donor photobleaching FRET and acceptor photobleaching FRET [4,6]. Some other approaches based on anisotropy [7,8], fluorescence lifetime [9,10], imaging spectroscopy [11], or lifetime imaging spectroscopy [12] require more specialized equipment, while yet others lack the quantitative measurement of FRET efficiency and rely on various FRET parameters that are usually made unreliable by the varying amounts and ratios of donor and acceptor in each examined pixel [13].

Donor photobleaching FRET, exploiting the decrease of excited state lifetime and consequential protection from photodestruction in the presence of FRET was the first quantitative approach applied to microscopy [14,15,20] and carries the advantage of being relatively simple to implement and rather sensitive, however, the need for external controls and the local variations in temperature and oxygenation can cause problems. The ratiometric approach based on coherent consideration of donor quenching, sensitized emission and cross-talk between channels was first applied in flow cytometry [1] and then adapted to microscopy [16]. While it yields itself readily to time-dependent measurements, the rather involved mathematics usually scares biologists away who then suffice with calculating dubious FRET ratios. A robust, easy to use, self-controlled FRET method, independent of donor and acceptor concentration and stoichiometry, is acceptor photobleaching FRET, which requires only simple image mathematics [4,17-19]. The de-quenching of the donor upon photodestructing the acceptor results in an increase of the donor fluorescence, which is proportional to the FRET efficiency *E*:

$$E=1-\frac{F_{Donor(quenched\_by\_acceptor)}}{F_{Donor(de\_quenched)}}$$

A measurement that exploits this proportionality is facilely implemented in confocal microscopy, thus providing the option of distinguishing various molecular association states even at the subcellular level. The method is also applicable to the ever-spreading family of green fluorescent protein (GFP) derivatives [20].

Image manipulation and analysis in biological research are often performed with the free ImageJ package [21]. In spite of the numerous plugins available, there are only three tools to help the evaluation of FRET. Two of them aid the assessment of ratiometric FRET images [22,23], while that provided by D. Stepensky has been the only freely available tool for acceptor photobleaching FRET [24]. This plugin allows the calculation of FRET efficiency based on average fluorescence signals (i.e. not on a pixel-by-pixel basis) from pixels above a pre-defined threshold in acceptor photobleaching images. It does not provide correction possibilities, registration, and it allows selection of rectangular shaped ROIs only. As the need for evaluating larger data sets for molecular interactions increases, we have undertaken to develop a program that addresses all the above deficiencies, and is also capable of quick semi-automatic processing of serial measurements.

## Implementation

The plugin was written in Java v1.6, and tested with ImageJ version 1.38×.

FRET efficiency *E*_(*i*, *j*) _is obtained pixel-by-pixel according to

$$E_{\left(i,j\right)}=1-\frac{\left(1-\alpha )(F_{D1(i,j)}-\delta F_{A1(i,j)}\right)}{\gamma \left(F_{D2(i,j)}-(\alpha \delta +(1-\alpha )\varepsilon )F_{A1(i,j)}\right)-\alpha \left(F_{D1(i,j)}-\delta F_{A1(i,j)}\right)}$$

where *F*_*D*1(*i*, *j*) _and *F*_*D*2(*i*, *j*) _are the donor fluorescence values of the pixel (*i*, *j*) before *(1) *and after *(2) *photobleaching the acceptor, and *F*_*A*1(*i*, *j*) _the acceptor fluorescence for the same pixel before photobleaching. All *F *values are background corrected throughout. *α*, *γ*, *δ *and *ε *are correction factors that are described below.

In some cases, photobleaching of the acceptor is not complete. As shown by van Munster et al. [25], the average FRET efficiency is directly proportional to the amount of available acceptor molecules assuming that photobleaching occurs indiscriminately to all acceptor molecules, and there is not more than one acceptor per donor molecule present. To correct for incomplete acceptor bleaching, the correction factor *α *is calculated as

*α *= ⟨*F*_*A*2(*i*, *j*)_/*F*_*A*1(*i*, *j*)_⟩

where *F*_*A*2(*i*, *j*) _and *F*_*A*1(*i*, *j*) _are intensities in the acceptor channel in pixels above threshold of the donor and acceptor labeled sample, before *(1) *and after *(2) *photobleaching.

The correction factor *γ *for unwanted photobleaching of the donor during the image acquisition procedure [4,26] can be calculated either as

*γ *= ⟨*F*_*Dd*1(*i*, *j*)_⟩/⟨*F*_*Dd*2(*i*, *j*)_⟩

or

*γ *= ⟨*F*_*Dd*1(*i*, *j*)_/*F*_*Dd*2(*i*, *j*)_⟩

where *F*_*Dd*1(*i*, *j*) _and *F*_*Dd*2(*i*, *j*) _are donor fluorescence intensities of donor only *(Dd) *samples in pixels above threshold before *(1) *and after *(2) *photobleaching the acceptor, and the ⟨⟩ signs denote mean value. Since FRET protects the donor from photobleaching [14], this factor calculated based on a sample labeled with donor only is not exactly accurate. However, the difference in practice is 10–20% of 1–2%, which may not cause great errors in determining FRET. Nevertheless, caution needs to be taken to minimize photobleaching of the donor during the measurement.

The program offers (also as for the correction factors *δ *and *ε*), the possibility to calculate the factor on a pixel-by-pixel basis and then average pixels above threshold for raw data, or, alternatively, to average the raw data for these pixels and then calculate an average correction factor.

In the case that the acceptor dye also fluoresces in the donor channel, FRET would be underestimated without correcting for this cross-talk [26]. The appropriate correction factor *δ *is calculated as

*δ *= ⟨*F*_*Da*1(*i*, *j*)_⟩/⟨*F*_*Aa*1(*i*, *j*)_⟩

or

*δ *= ⟨*F*_*Da*1(*i*, *j*)_/*F*_*Aa*1(*i*, *j*)_⟩

where *F*_*Da*1(*i*, *j*) _and *F*_*Aa*1(*i*, *j*) _are signals in the donor and acceptor channels in pixels above threshold of an acceptor only labeled sample, before *(1) *photobleaching the acceptor.

In some cases, photobleaching the acceptor can yield a photoproduct with distinct absorption and emission properties, which can contribute to the post-bleach donor signal, resulting in the overestimation of FRET efficiency [4]. The correction factor *ε *for such acceptor-photoproduct is calculated as

*ε *= ⟨*F*_*Da*2(*i*, *j*)_⟩/⟨*F*_*Aa*1(*i*, *j*)_⟩

or

*ε *= ⟨*F*_*Da*2(*i*, *j*)_/*F*_*Aa*1(*i*, *j*)_⟩

where *F*_*Da*2(*i*, *j*) _and *F*_*Aa*1(*i*, *j*) _are intensities in the donor and acceptor channels in pixels above threshold of an acceptor only labeled sample, before *(1) *and after *(2) *photobleaching.

The calculation of the constants *γ*, *δ *and *ε *requires taking images with the same photobleaching protocol on samples labeled with donor only and acceptor only, which usually need to be taken anyway. To correct for shifts in the x-y plane, the images are registered using the Fast Hartley Transform algorithm [27] implemented in the ImageJ package. All corrections are optional and can be activated/inactivated in the "Corrections" menu of the plugin.

## Results

With our program AccPbFRET, which can be found in the additional file [see Additional file 1] or can be downloaded from its homepage [28], FRET efficiencies are calculated pixel-by-pixel, and their distribution is determined for any user defined rectangular, polygonal, or freehand type ROI or subcellular location. Accurate selection of the examined cellular components is furthered by the provision to interactively set threshold values of donor and, optionally, acceptor fluorescence intensities, and to also gate using images with relevant independent fluorescent labels in the same sample. In addition, our plugin provides automatic registration of the images, an absolute necessity for perfect alignment of donor images taken before and after photobleaching. An example analysis with and without registration (along with other examples) can be found in the additional file [see Additional file 1]. We compared the results of the same images obtained with FRETcalc [24] and AccPbFRET, and we obtained similar FRET efficiencies, 15.1% and 14.7%, respectively. However, when we used images that needed registration because of a few pixels shift, the results changed to 5.7% versus 14.6%.

Other important issues with acceptor photobleaching FRET is correcting for bleaching of the donor, for the cross-talk of the acceptor and/or its provisional photoproduct to the donor channel, and partial photobleaching of the acceptor, which are also solved by AccPbFRET.

**Figure 1:**
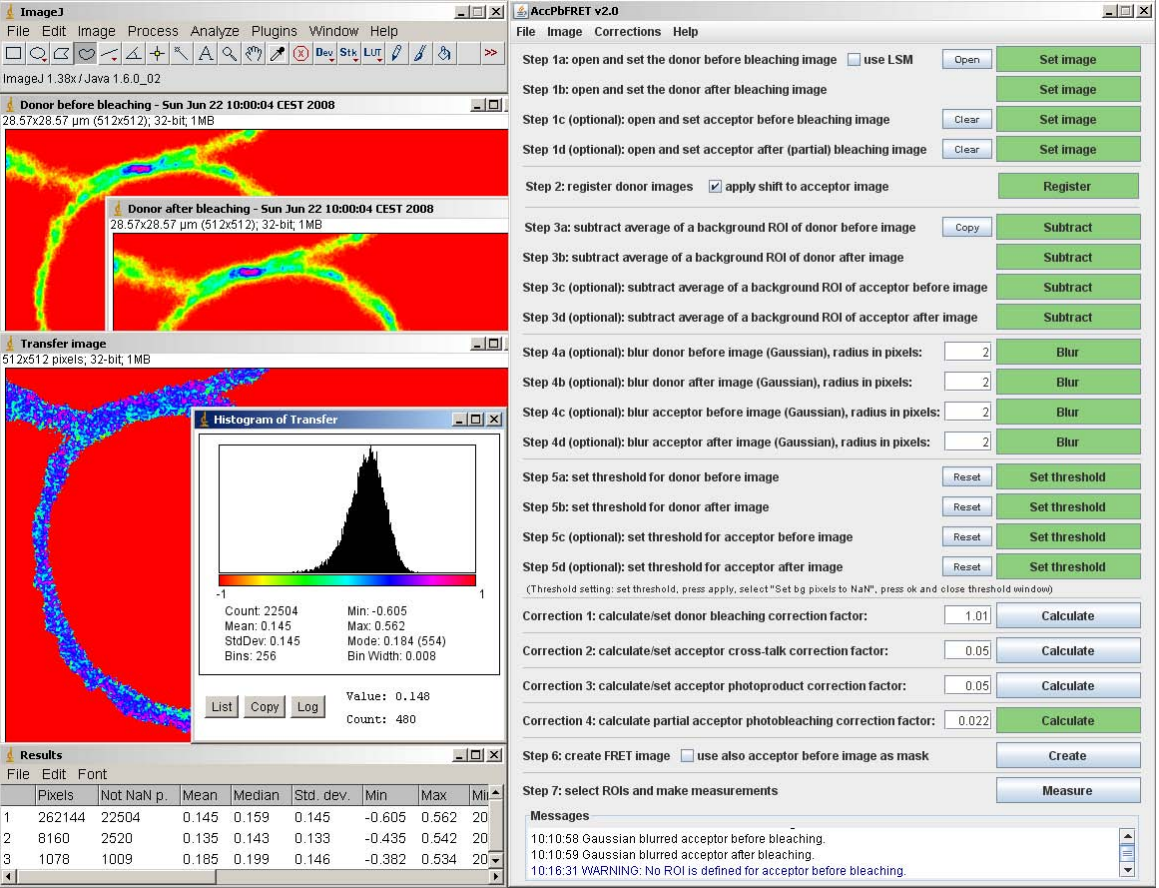
**Screenshot of an example analysis with the AccPbFRET plugin**. The ImageJ (left top) and AccPbFRET (right) dialog windows are displayed, together with donor channel source images taken by a confocal microscope (labeled appropriately as 'Donor before bleaching', 'Donor after bleaching'), as well as the calculated, corrected FRET image ('Transfer image'), on which ROIs can be selected and statistics calculated. These statistics can be seen in a separate 'Results' window, and a histogram of the FRET distribution is also presented.

The steps of creating the FRET image are enumerated in the main program window, so the user only needs to follow the instructions (see image of main window on the right side of Figure 1 for details). Supplementary information appears as a tooltip when the mouse pointer is hovered over an option or button that might need further explanation. Thus even novice users, those unfamiliar with FRET and/or with Java and ImageJ can quickly go through the analysis procedure, without the danger of committing the usual errors. On average, the complete analysis from loading the images to arriving at reliable results takes less than a minute.

**Figure 2:**
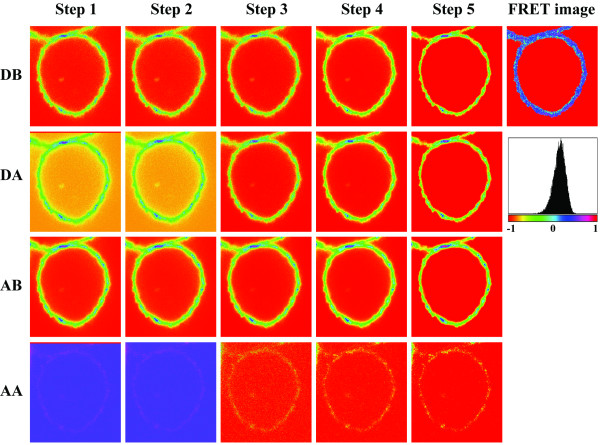
**The analysis process**. In this figure, the changes of donor and acceptor images during the steps of the analysis process are shown. DB, DA: donor images before and after bleaching the acceptor; AB AA: acceptor images before and after bleaching (same images as in Figure 1, cropped to fit the page). STEP1: original images; STEP2: images after registration (note the disappearance of the top lines from DA and AA); STEP3: after background subtraction; STEP4: after Gaussian filtering; STEP 5: thresholded images. The corrected FRET/transfer image and histogram derived from it are also displayed.

Figure 2 shows the images as they evolve through the analysis process to finally yield the FRET ("transfer") image. As a biological example, intramolecular FRET characteristic of receptor conformation was measured among two cell surface ErbB2 tyrosine kinase epitopes on SK-BR-3 cells using a 4-channel CLSM. The two epitopes were labeled specifically by the fluorescently tagged antibodies rHu4D5 (trastuzumab) and 2C4. Confocal imaging was carried out with a Zeiss (Göttingen, Germany) LSM 510 confocal laser scanning microscope (CLSM) using a Plan-Apochromat 63×/NA 1.4, oil DIC objective. The donor, AlexaFluor 555 was excited with a 543-nm HeNe laser and detected through a 560–615 nm emission filter. As acceptor, Cy5 was excited with a 633-nm HeNe laser and detected through a 650 nm longpass filter. The panels in the four consecutive rows depict donor (D) and acceptor (A) channel images before (DB, AB) and after (DA, AA) photobleaching the acceptor. The columns show the original images (step 1); images after registration (step 2); after background subtraction (step 3); after Gaussian filtering (step 4); and finally the thresholded images (step 5). Correction factors are obtained using a similar algorithm. The corrected FRET/transfer image is then calculated, and the histogram derived from it is also displayed. The measurement in this case reveals a mean FRET efficiency in the cell membrane of 14.5%, indicating that the extended dimerization loop of ErbB2 is in proximity of the juxtamembrane domain. Such measurements can be specific enough to support molecular modeling as was demonstrated in the case of the nearly full length ErbB2 earlier [29].

When evaluating large amounts of molecular interaction data on several hundreds of image sets, the optional semi-automatic mode allows nearly three times faster processing relative to the single-image mode. In semi-automatic processing mode (tested with Zeiss LSM 510 Version 4.0), the program opens images sequentially in the chosen directory, and only threshold setting(s) and creation of the FRET image need to be done manually. The upper left corner (1/6 × 1/6 of the image) is considered automatically as background.

## Conclusion

The AccPbFRET plugin provides an easy to use graphical interface, which leads the user through the evaluation process, and does not require cumbersome pre-setting of various parameters. It allows correcting for bleaching of the donor, for the cross-talk of the acceptor and its photoproduct to the donor channel and for partial acceptor photobleaching. Furthermore, automatic registration and semi-automatic analysis of large image sets is provided, which are not available in any existing evaluation software.

## Availability and requirements

• Project name: AccPbFRET

• Project home page: 

• Operating system(s): platform independent

• Programming language: Java

• Other requirements: ImageJ 1.38× (bundled with Java 1.6.0_02) or higher, screen resolution 1280 × 900 or higher

• License: free software

• Any restrictions to use by non-academics: none

## Authors' contributions

GV conceived and coordinated the project. JR, JS and GV participated in software design and testing. JR implemented, and JR and GV tested the software. JR drafted the manuscript, JS and GV helped to create the final version. All authors read and approved the final manuscript.

## Supplementary Material

Additional file 1 **Source code and example images**. This file contains the AccPbFRET.java source code and some example LSM and TIFF image files together with explanations. Click here for file
