# Supplementary figures and images for: AccPbFRET: An ImageJ plugin for semi-automatic, fully corrected analysis of acceptor photobleaching FRET images
Source: BMC Bioinformatics. 2008 Aug 19;9:346. doi: 10.1186/1471-2105-9-346 (PMC2571114; doi:10.1186/1471-2105-9-346)

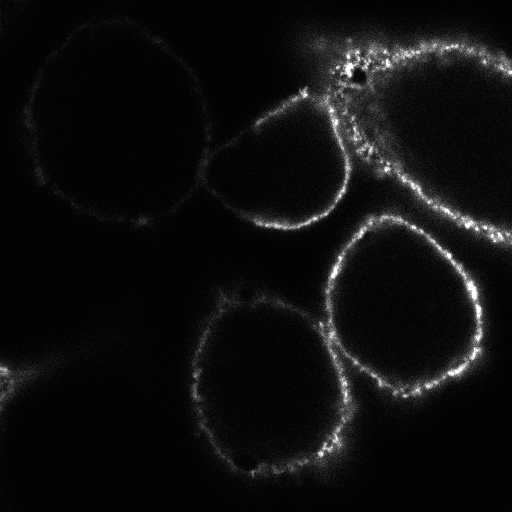

Supplement: Additional file 1 — Source code and example images. This file contains the AccPbFRET.java source code and some example LSM and TIFF image files together with explanations. [file 1471-2105-9-346-S1.zip › AccPbFRET_v2_0/3-partial-bleaching-example/acceptor-after-bleaching.tif]

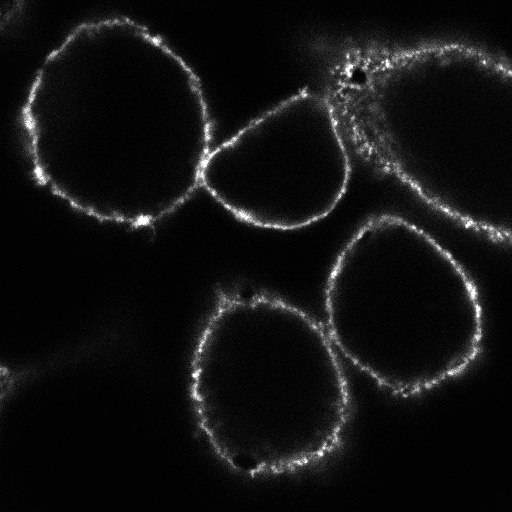

Supplement: Additional file 1 — Source code and example images. This file contains the AccPbFRET.java source code and some example LSM and TIFF image files together with explanations. [file 1471-2105-9-346-S1.zip › AccPbFRET_v2_0/3-partial-bleaching-example/acceptor-before-bleaching.tif]

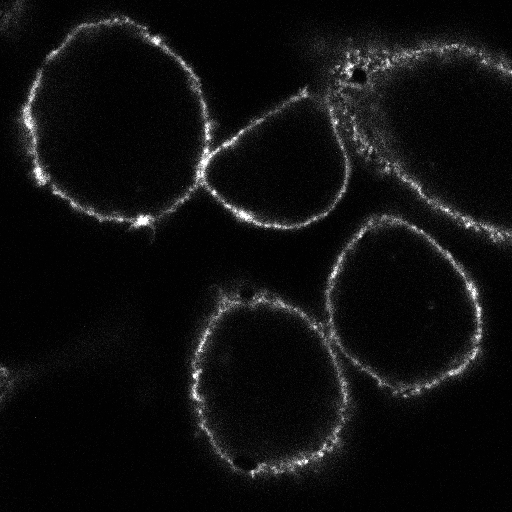

Supplement: Additional file 1 — Source code and example images. This file contains the AccPbFRET.java source code and some example LSM and TIFF image files together with explanations. [file 1471-2105-9-346-S1.zip › AccPbFRET_v2_0/3-partial-bleaching-example/donor-after-bleaching.tif]

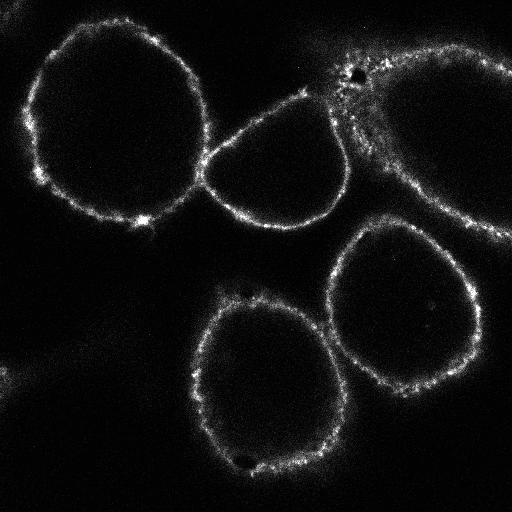

Supplement: Additional file 1 — Source code and example images. This file contains the AccPbFRET.java source code and some example LSM and TIFF image files together with explanations. [file 1471-2105-9-346-S1.zip › AccPbFRET_v2_0/3-partial-bleaching-example/donor-before-bleaching.tif]
